# Multiple Kernel Synthesis of Head CT Using a Task-Based Loss Function

**DOI:** 10.1007/s10278-023-00959-x

**Published:** 2024-01-12

**Authors:** Brandon J. Nelson, Daniel G. Gomez-Cardona, Jamison E. Thorne, Nathan R. Huber, Lifeng Yu, Shuai Leng, Cynthia H. McCollough, Andrew D. Missert

**Affiliations:** 1 Department of Radiology, Mayo Clinic, 200 First Street SW, 55905 Rochester, MN USA; 2 Department of Imaging, Gundersen Health System, La Crosse, WI USA

**Keywords:** Loss function, CNN, Denoising, CT, Head, Neuro

## Abstract

In CT imaging of the head, multiple image series are routinely reconstructed with different kernels and slice thicknesses. Reviewing the redundant information is an inefficient process for radiologists. We address this issue with a convolutional neural network (CNN)-based technique, synthesiZed Improved Resolution and Concurrent nOise reductioN (ZIRCON), that creates a single, thin, low-noise series that combines the favorable features from smooth and sharp head kernels. ZIRCON uses a CNN model with an autoencoder U-Net architecture that accepts two input channels (smooth- and sharp-kernel CT images) and combines their salient features to produce a single CT image. Image quality requirements are built into a task-based loss function with a smooth and sharp loss terms specific to anatomical regions. The model is trained using supervised learning with paired routine-dose clinical non-contrast head CT images as training targets and simulated low-dose (25%) images as training inputs. One hundred unique de-identified clinical exams were used for training, ten for validation, and ten for testing. Visual comparisons and contrast measurements of ZIRCON revealed that thinner slices and the smooth-kernel loss function improved gray-white matter contrast. Combined with lower noise, this increased visibility of small soft-tissue features that would be otherwise impaired by partial volume averaging or noise. Line profile analysis showed that ZIRCON images largely retained sharpness compared to the sharp-kernel input images. ZIRCON combined desirable image quality properties of both smooth and sharp input kernels into a single, thin, low-noise series suitable for both brain and skull imaging.

## Background

X-ray computed tomography (CT) is one of the most widely used and clinically impactful imaging modalities, with over 80 million CT exams performed annually in the USA [1]. Due to increasing patient loads and technological advances, radiologists are often faced with the challenge of interpreting multiple CT image series in a very limited amount of time. Considering just a single CT series can consist of hundreds or even thousands of detailed slices of a 3D volume, this quickly leads to information overload. Information overload can hinder efficiency and contribute to reader fatigue [2–6]. One of the main reasons for needing multiple image series for a single exam is the well-known trade-off between spatial resolution and noise that is primarily determined by the reconstruction kernel. Thus, radiologists must typically review a smooth-kernel (low-noise) series for low-contrast features, and sharp-kernel (high-noise) series for small-scale and high-contrast features. Achieving both smooth and sharp characteristics in a single series cannot be done today with traditional reconstruction methods.

Several approaches have been proposed to increase the efficiency of CT image viewing by combining multiple task-specific series into a single series that is appropriate for multiple imaging tasks, a process referred to hereafter as multi-kernel synthesis [7–12]. Hounsfield unit (HU) threshold-based synthesis techniques consist of replacing image pixels obtained with smooth kernels and corresponding to high-contrast bone anatomy with sharp-kernel pixels, producing a single image with matched diagnostic performance as the two individual kernel images [10, 11]. Other approaches utilizing iterative reconstruction avoid boundary discontinuities by spatially varying their regularization function over different anatomy to control the local smoothness, but at the cost of increased computation time [8]. A more recent approach to multi-kernel synthesis used a convolutional neural network (CNN) denoising model with multiple input channels yielding improved noise reduction and sharpness preservation [9].

While CNN-based kernel synthesis was shown to be promising for abdominal imaging applications, CT imaging of the head poses additional challenges that are specifically addressed in this work. Compared to routine abdominal imaging, head imaging requires a wider range of sharp and smooth kernels, each having specialized post-processing. Reconstruction kernels offering high-spatial resolution and edge enhancement are routinely used to assess fractures in the skull. On the other hand, low noise is needed to detect the small HU differences relevant to brain imaging, such as between gray and white matter. To improve gray-white matter differentiation and aid in the detection of low-contrast lesions, smooth reconstruction kernels specific for the head sometimes include additional post-processing steps [13]. Additionally smooth-kernel image series are reconstructed with greater slice thickness to compensate for noise, which reduces the visibility of small low-contrast features due to partial volume averaging.

To account for this and other customized kernel features for head imaging, a new framework, synthesiZed Improved Resolution and Concurrent nOise reductioN (ZIRCON), is introduced, expanding upon previous CNN-based multi-kernel synthesis methods [9]. ZIRCON is a denoising and image synthesis CNN-model that uses a unique loss function with two complementary loss terms to parameterize training. In this exploratory study, we introduce ZIRCON and evaluate the ability to reduce noise and enhance soft-tissue contrast in the brain while preserving sharp details in the skull, effectively combining the favorable image quality features of each input kernel into a single image series optimized for imaging of the head.

## Methods

### Methodology

**Fig. 1:**
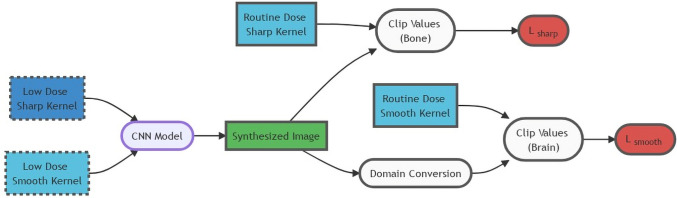
Summary of ZIRCON task-based loss training method. Smooth- and sharp-kernel low-dose images are given as model training inputs. After passing through a denoising U-Net CNN model, pixels in the range of brain HUs ($0 HU < z\left(\overrightarrow{r}\right)<80 \mathrm{HU})$ are smoothed via domain conversion before calculating ${L}_{smooth}$ from the matching routine-dose smooth-kernel image pixels. Pixels outside the brain HU range are compared to the matching routine-dose sharp-kernel image pixels, ${L}_{sharp}$. (ZIRCON = synthesiZed Improved Resolution and Concurrent nOise reduction; CNN = convolutional neural network; HU = Hounsfield unit)

A schematic diagram of the ZIRCON model optimization framework is illustrated in Fig. 1. In the training phase, simulated low-dose (LD) images from a smooth kernel and a sharp kernel are concatenated along the channel dimension and passed to a CNN model based on the U-Net architecture [14]. The CNN produces a single output image corresponding to a synthesized image with both low noise and high spatial resolution. The synthesized image is compared to the corresponding routine-dose (RD) images using a task-based loss function consisting of two terms, and the model weights are updated using a variant of stochastic gradient descent. Training and evaluation of this framework was performed using retrospective data. Details regarding the data generation, model, loss functions, and evaluation methods are described in the following sections.

### Data Preparation

A dataset consisting of CT images of patients with suspected head trauma was collected retrospectively from exams performed in August and September 2020 to demonstrate the clinical utility of the ZIRCON framework under a protocol approved by our Institutional Review Board. Inclusion criteria considered patients who underwent head CT exams in the emergency department for trauma or acute-onset symptoms suspected of fracture, intracranial hemorrhage, and/or infarction. A total of 585 cases were collected. Screening by a radiologist fellow revealed acute findings in 82 patients (hemorrhage ($N=52$), hemorrhagic brain infarction ($N=2$), infarction ($N=15$), metastasis ($N=2$), fracture ($N=6$), and both fracture and hemorrhage ($N=5$)). Screening was performed comparing the clinical CT report, and imaging findings, as well as any prior and subsequent imaging exams and reports in the electronic medical record. This study was approved by an Institutional Review Board, is compliant with standards from the Health Insurance Portability and Accountability Act (HIPAA), and all patients permitted use of their clinical records for research purposes.

These exams were performed as part of routine patient care using commercially available CT scanners (SOMATOM Force, Siemens Healthineers GmbH, Forchheim, Germany) and a protocol specific to head trauma: no contrast agent, 1-s rotation, $192\times 0.6$ mm collimation, 0.6 pitch, 120 kV tube potential, 350 effective mAs, 250 mm reconstructed field of view, and no tube current modulation with a CTDIvol of 49.7 mGy. A previously validated projection-domain noise-insertion tool [15] was used to simulate exams with a dose level corresponding to 25% of the routine dose. Multiple CT image series were reconstructed and deidentified using a dedicated offline reconstruction workstation (ReconCT, Siemens Healthineers) to create the training dataset and the clinical reference images (Table 1). The training images were reconstructed using weighted filtered backprojection, whereas the clinical reference images were reconstructed using iterative reconstruction with a medium strength setting.

**Table 1:** Image reconstruction settings and noise levels used for generating the image series used in this study. The noise levels were measured within a 2.4 cm^2^ uniform region of the vitreous body in a representative case from the training data

*Series description*	*Kernel*	*Thickness [mm]*	*Increment [mm]*	*Relative dose [%]*	*Approx. noise level [HU]*
Smooth LD	Hr40	0.75	0.7	25	12.8
Sharp LD	Qr69	0.75	0.7	25	80.1
Smooth RD	Hr40	0.75	0.7	100	6.0
Sharp RD	Qr69	0.75	0.7	100	34.5
Smooth RD reference	Hr40	5.0	5.0	100	3.2

*LD* low does, *RD* routine dose

From this dataset, 110 exams from unique patients were randomly selected for development of the ZIRCON framework, including 100 for model training and 10 used to monitor for overfitting and for hyperparameter tuning (validation), with the remaining cases were reserved for testing and future studies. The training and validation partition contained a mix of normal (*N* = 94) and abnormal (*N* = 16) cases. The training data was randomly cropped into 300,000 image patches with a size of 64×64 pixels. No augmentation or other normalization was applied to the data a priori, although the proposed U-Net [14] model included a preprocessing layer described in the next section. The training inputs consisted of paired smooth-kernel and sharp-kernel images patches from the simulated quarter dose data, with the “labels” being the corresponding routine dose patches. Although training was performed on image patches, a fully convolutional model was used, so inference was performed directly on full-size (512×512) images.

### Model Optimization

#### Network Architecture

**Fig. 2:**
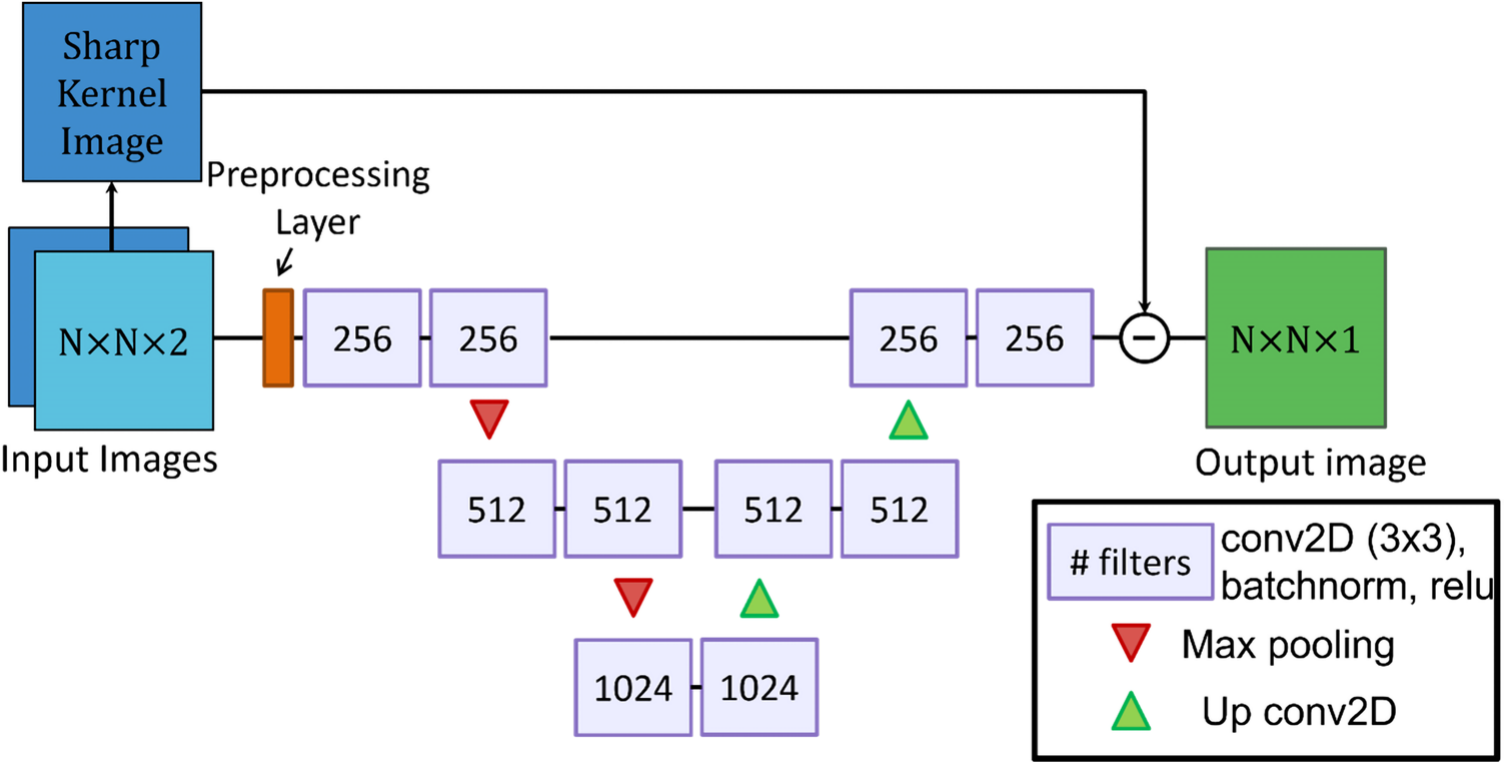
Network architecture of the residual CNN kernel synthesis model, “CNN Model,” from Fig. 1. Input and output images display the array dimensions for height, width, and channels, respectively. The numbers in each convolutional block denote the number of filters used (CNN = convolutional neural network). The minus symbol denotes pixel-wise subtraction

A residual U-Net [14] variant with randomized initial weights (Glorot initialization) was chosen as the CNN architecture for this study. A CT-specific preprocessing layer was added prior to the first convolutional layers that generated normalized feature maps based on standard clinical CT window settings for soft tissue, bone, and brain imaging. This preprocessing layer subtracted the window level for each pixel and then divided by the window width, with the outputs for each window setting concatenated in the channel dimension. These features were then fed into a shallow U-Net with residual blocks (Fig. 2). Network implementation and optimization was performed using Tensorflow [16]. Optimization was performed over 100 epochs with the Adam optimizer and a step-decay learning rate scheduler with a starting rate of 0.001, a decay factor of 0.25, and 3 decay steps over the training period. The batch size used during training was 26.

#### Task-Based Loss Function

The choice of loss function is critical to ensuring that the CNN achieves the desired mixing of features from the smooth-kernel and sharp-kernel images. In this work, we propose a “task-based” loss function that consists of the sum of two terms: ${L}_{sharp}$ and ${L}_{smooth}.$

The decomposition of the loss function into two terms related to specific diagnostic tasks encourages the model to preserve image features relevant for both tasks in the output image. Thus, ${L}_{sharp}$ should emphasize high-resolution features for visualizing the bones of the skull, whereas ${L}_{smooth}$ should emphasizes the low-contrast features that are critical for diagnostic brain imaging.

The task-based loss terms used in this work were empirically defined as follows:

1 $${L}_{sharp}=\alpha {D}_{sharp}{\left|\left|z-{x}_{sharp}\right|\right|}_{2}$$

2 $${L}_{smooth}=\beta {D}_{smooth}\left({\left|\left|C\left(z,\sigma \right)-{x}_{smooth}\right|\right|}_{2}+\gamma TV\left(z\right)\right)$$

Here, $z$ denotes the CNN output image, ${x}_{sharp}$ denotes the RD sharp-kernel reference image, and ${x}_{smooth}$ denotes the RD smooth-kernel image. The symbols ${D}_{sharp}$ and ${D}_{smooth}$ represent pixel-wise weighting factors that are derived from the HU values in the sharp-kernel and smooth-kernel images, respectively. The operation $C\left(z,\sigma \right)$ denotes convolution with a Gaussian kernel with a standard deviation of $\sigma .$ TV corresponds to the total variation. The global scaling parameters$\alpha$,$\beta$, and $\gamma$ determine the relative strength of each term. All multiplication is assumed to be element-wise and $||.||_{2}$ is the ${L}_{2}$ norm. The parameter values used for this study are as follows: $\alpha =0.5$,$\beta =7.0$,$\gamma =1{e}^{-5}$, and $\sigma =0.47$ mm.

The pixel-wise weighting factors ${D}_{sharp}$ and ${D}_{smooth}$ were calculated using binary thresholding with distance transformations to smooth the boundaries between regions. First a binary mask was created to mask out pixels outside of the range $\left[0, 80\right]$ HU $.$ A distance transform based on the work of by Dorn et al. [7] was used to generate a weighting mask defined by the minimum Euclidean distance to pixels outside of the mask. This distance transformed mask $D(\overrightarrow{r})$ was then truncated and normalized:

3 $${D}_{trunc}\left(\overrightarrow{r}\right)=\left\{\begin{array}{c}1, if D\left(\overrightarrow{r}\right)>d\\ {(D(\overrightarrow{r})/d)}^{p}, else\end{array}\right.$$

The width of the transition between regions is defined by $d$ and the rate of transition between regions is controlled by power $p$. By using smooth transitions between regionally weighted loss functions different image quality properties can be locally optimized while avoiding boundary discontinuity artifacts [11]. For the current study a transition width of $d\ge 7$ pixels were found to be sufficient. A relatively steep transition achieved with a cubic transition ($p=3$) was chosen to minimize loss in sharpness at the skull boundary while avoiding transition artifacts. ${D}_{sharp}$ and ${D}_{smooth}$ then derive from this smoothed weighting mask

4 $${D}_{sharp}\left(\overrightarrow{r}\right)={D}_{trunc}\left(\overrightarrow{r}\right)$$

5 $${D}_{smooth}\left(\overrightarrow{r}\right)=1-{D}_{trunc}(\overrightarrow{r})$$

### Model Evaluation

The ideal output of the ZIRCON model is a single, low-noise, high-spatial resolution image series that can be used to perform diagnostic tasks that would otherwise require multiple series with different reconstruction parameters. Noise magnitude in ZIRCON images was measured as the standard deviation in three regions-of-interest (ROIs) in the brain that were selected by the authors to have approximately uniform intensity. These ROIs were from a random patient in the reserved test data and contained no pathology. Noise magnitude in these ROIs was then compared to that obtained with the corresponding smooth-kernel reconstructions. Soft-tissue contrast in the ZIRCON images was measured as the absolute difference in HUs between white-matter ROIs directly adjacent to gray-matter ROIs. These measurements were then used to calculate the contrast-to-noise ratio (CNR) between gray- and white-matter. Spatial resolution was evaluated by comparing line profiles for the ZIRCON images and clinical sharp-kernel images across relevant small-scale features such as skull fractures. Finally, the presence of clinically relevant artifacts, such as skull blooming that can mimic subarachnoid hemorrhage, was assessed by visual inspection of pathological ROIs and review with a trained radiologist fellow. These artifacts are commonly seen in low dose head CT images obtained through iterative reconstruction, which is inherent to the training data set used in this work.

## Results

**Fig. 3:**
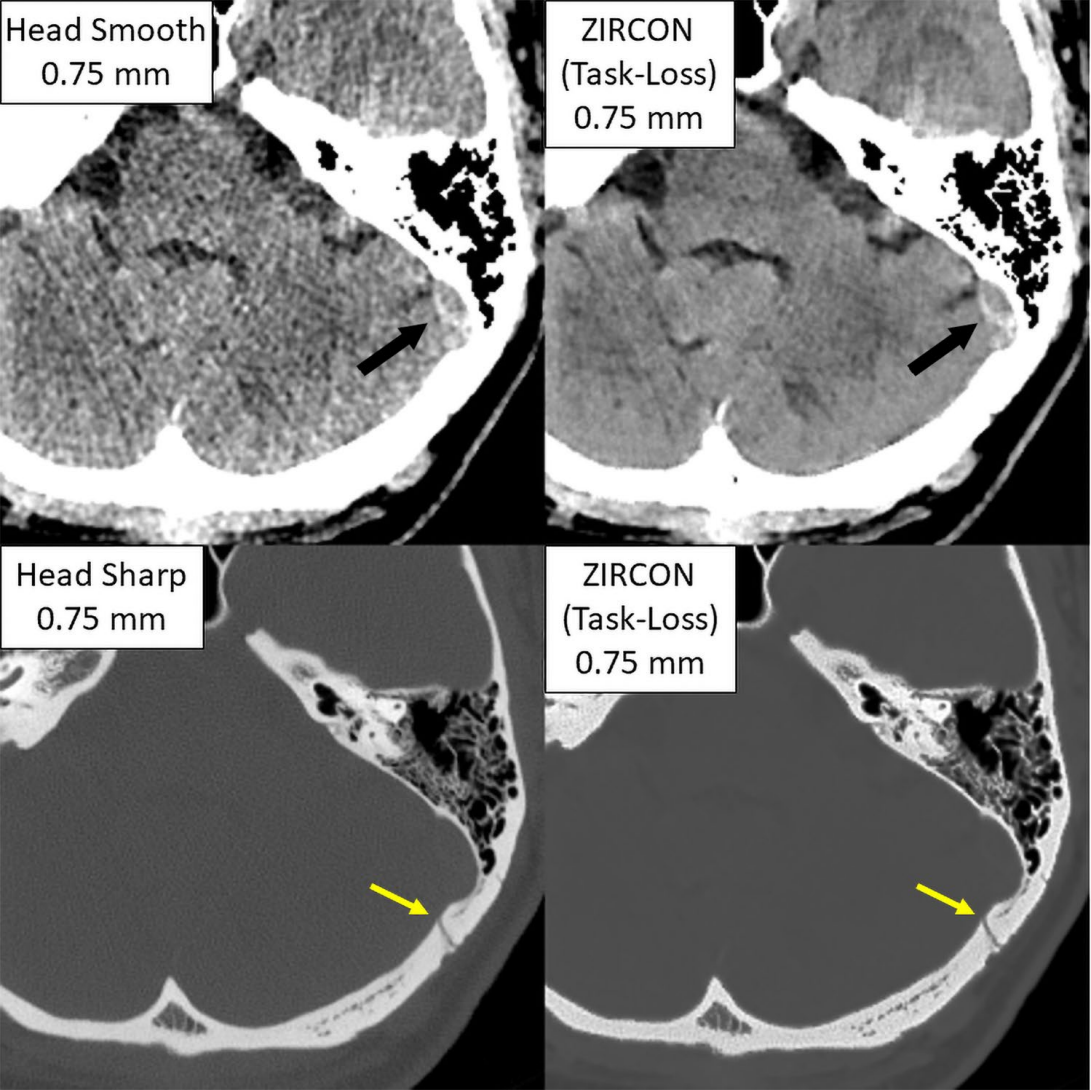
Visual comparison between both input image series (head smooth and head sharp kernels) vs ZIRCON’s single output. The smooth kernel input is shown with brain display settings (ww/wl: 80/40 HU) while the sharp kernel input is shown with bone display settings (ww/wl: 2800/600 HU). The arrow in the smooth kernel image identifies a potential hemorrhage while the arrow in the sharp kernel image identifies a squamosal suture (ZIRCON = synthesiZed Improved Resolution and Concurrent nOise reduction; HU = Hounsfield unit)

The qualitative performance of ZIRCON is demonstrated in Fig. 3. Compared to the input images, ZIRCON features the low noise levels of the smooth kernel images while exhibiting the high spatial resolution of the sharp-kernel images. As exhibited in the figure, these factors improve the conspicuity of a hemorrhage while also maintaining resolution of fine anatomic details. Together this combination of thin slice, low noise, and enhanced soft tissue contrast enhancement in the brain along with preserved sharpness in the skull demonstrates the primary benefit of ZIRCON as these image quality properties are usually mutually exclusive in a single image series.

**Fig. 4:**
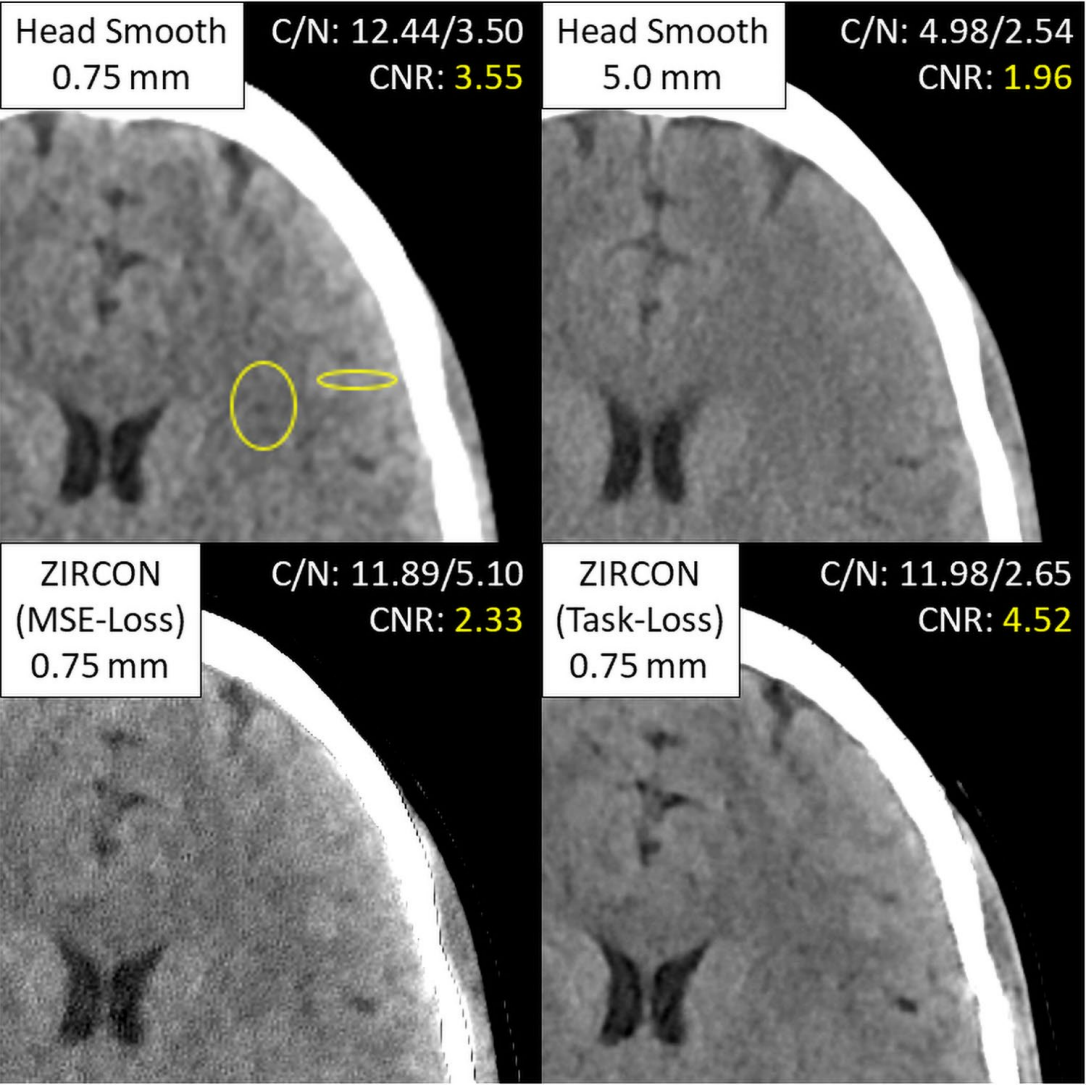
Comparison of soft tissue performance between thin and thick smooth kernel image series, previous kernel synthesis model trained with generic MSE loss and the proposed ZIRCON model with a Task-based loss (ww/wl: 80/40 HU). ROIs used to extract contrast (C) and noise (N) measurements as well as the resulting CNR between gray and white matter are also displayed for each case (ZIRCON = synthesiZed Improved Resolution and Concurrent nOise reduction; HU = Hounsfield unit; ROI = region of interest; CNR = contrast-to-noise ratio)

These image quality improvements can be attributed to the task-based loss function defined in Eqs. 1 and 2. Notably, the addition of ${L}_{smooth}$ in the task-based loss function improves noise reduction and soft-tissue contrast compared to optimizing with a general MSE loss (the “ZIRCON-MSE” series in Fig. 4). Additionally, the task-based loss function further reduces artifacts arising from the commercial sharp-kernel reconstructions, such as the edge overshoot near the skull boundary seen in the bottom left panel in Fig. 4. Contrast measurements from elliptical ROIs in adjacent regions of gray and white matter show comparable contrast between ZIRCON and the thin smooth series (12.44 vs 11.98, respectively) suggesting that ${L}_{smooth}$ was effective in learning the gray-white matter contrast enhancement. When combined with lower noise, shown by standard deviation measurements (3.50 vs 2.65), this results in an overall higher gray-matter white-matter CNR in ZIRCON Task-Loss images.

**Fig. 5:**
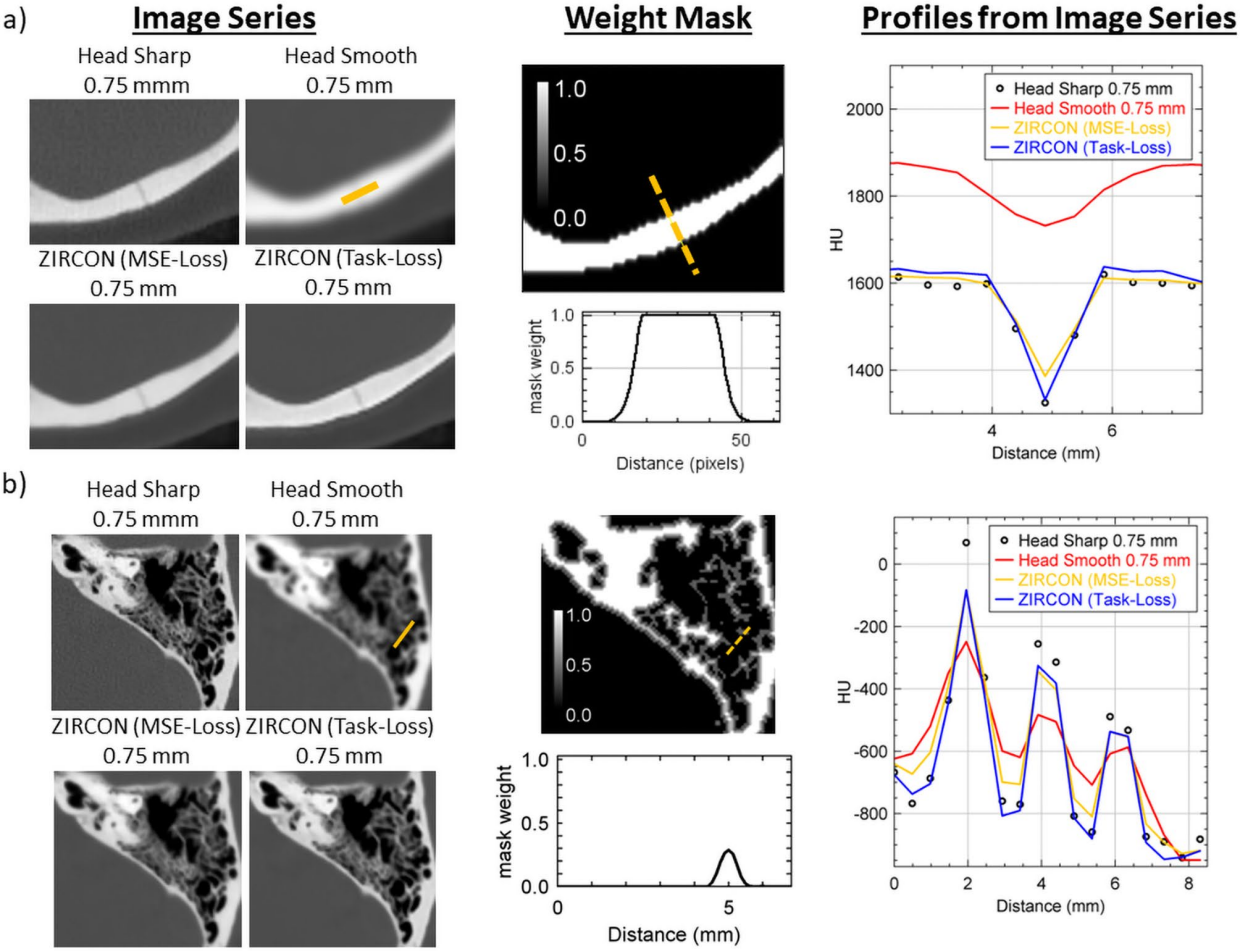
Comparison of bone sharpness for different ZIRCON models compared to the sharp and smooth inputs. “ZIRCON (MSE)” is the ZIRCON denoising model trained with a generic mean-squared-error loss function while “ZIRCON (Task-Loss)” is the output immediately following the CNN denoising model summarized in Fig. 2 and trained with the proposed task-based loss function Eqs. (1) and (2). **a** Line profile comparison covering a small fracture in a cortical bone region of the skull between different image series being investigated. The center columns show the mask used in the loss function. The mask weight is saturated in the bone region meaning that it is replaced with the sharp input kernel with a blended transition near the boundary where the model output is progressively added. **b** In the trabecular region of the temporal bone, the lower intensity bone structures are missed by the segmentation mask and are therefore subject to smoothing by the model (ZIRCON = synthesiZed Improved Resolution and Concurrent nOise reduction; CNN = convolutional neural network)

The performance of ZIRCON in preserving high-frequency details was assessed via line profile comparisons along different regions in the skull. Figure 5a compares the performance of the different image series at resolving an occipital fracture. When trained with generic MSE loss, “ZIRCON (MSE) shows unsatisfactory sharpness compared to the sharp input kernel. After incorporating the task-based loss function, “ZIRCON (Task Loss)”, there is evidence of preserved sharpness. This holds true where the masks correctly distinguish between soft and bone tissue. In Fig. 5a, the visual sharpness is preserved between the input sharp kernel and the ZIRCON (Task Loss) whereas in Fig. 5b, the visual sharpness of ZIRCON (Task Loss) in the temporal bone region is limited by the ability to segment these lower intensity structures. The latter remains a challenge in ZIRCON’s current intensity threshold-based segmentation.

## Discussion

Diagnostic CT imaging for head trauma is a challenging clinical scenario that places high demands on image quality. The conflicting requirements of low noise and high spatial resolution typically requires reviewing multiple image series with different reconstruction parameters to get a complete understanding of the patient’s medical condition. If the diagnostically relevant image features could be combined into a single low-noise, thin-slice, sharp-kernel image series, the workflow efficiency for the radiologist could be significantly improved.

Bridging the gap between these image quality constraints presents a challenging task. The noise level of the sharp-kernel reconstructions must be reduced by a factor of at least 16 while also maintaining both spatial resolution and soft-tissue contrast. The results described in the “Results” section demonstrate that the ZIRCON framework generally meets these objectives. Through noise-regulated optimization with a task-based loss function, a CNN model was trained to process multiple image series with different reconstruction parameters and synthesize a single output image exhibiting the clinically relevant features. This opens the possibility of reducing the number of image series that need to be interpreted for complex exams. Additionally, the reduced noise levels in the synthesized image series provide enhanced low contrast performance due to reduced partial volume effects in the thin-slice images.

The focus of this work is to present a proof-of-concept framework for training CNN models to produce extremely low-noise, high-resolution CT images. The proposed ZIRCON image synthesis method is not iterative and thus has reduced computation time at inference time compared to previous iterative reconstruction-based image synthesis. Additionally, by incorporating loss functions informed by the unique imaging requirements of head CT imaging, ZIRCON can achieve improved sharpness in bone and better noise reduction and texture in the brain than previous CNN-based image synthesis (ZIRCON-MSE). Although we demonstrate this technique as promising when applied to routine-dose head CT exams, additional studies are required to further investigate the clinical impact of different noise levels and potential limitations. Precise evaluation of the diagnostic performance of the ZIRCON images will be explored in future publications. A preliminary radiologist evaluation of 50 head CT exams with a mix of pathologies in the brain (infarction, hemorrhage, mass) and bone (fracture) found the single ZIRCON series had similar diagnostic performance (in terms of jackknife free-response receiver operating characteristic or JAFROC) compared to multiple series with commercial reconstruction [17].

We also note some practical and technical limitations that should be further explored. In terms of generalizability, we expect the ZIRCON models to behave similar to other AI-based denoising models. Previous studies [18, 19] have shown that such models generalize well across different noise/dose levels but are susceptible to out-of-distribution examples reconstructed with significantly different kernels. Additionally, a key technical limitation is the threshold-based segmentation used to create the pixel-wise weighting factors ${D}_{sharp}$ and ${D}_{smooth}$. When high-quality segmentations are not available, as is often the case in temporal bones or sinuses of the head, the task-based loss function does not significantly differ from the standard MSE-trained model. We hypothesize that implementing an auxiliary CNN model to perform semantic segmentation of these complex anatomic regions may result in further improvements. As the focus of this work was on investigating the use of new loss functions to synthesize CT image series and not on identifying the optimal skull segmentation technique, investigating alternative and more advanced segmentation techniques remains as future work.

## Conclusion

Given the exponential rise in the number and complexity of CT imaging exams, there is a demonstrated need for technology that reduces information overload for the interpreting radiologist. In this work, we propose multiple-kernel synthesis as a way of addressing this issue by generating images that more efficiently convey the clinically relevant information, thereby simplifying the workflow for the radiologist.

## Declarations

### Presentation at Meeting

Selected sections were presented during oral presentation at AAPM 2021 and RSNA 2021.
